# Ultrasonography and color Doppler of proximal gluteal enthesitis in juvenile idiopathic arthritis: a descriptive study

**DOI:** 10.1186/1546-0096-9-22

**Published:** 2011-08-11

**Authors:** Louise Laurell, Michel Court-Payen, Susan Nielsen, Marek Zak, Carsten Thomsen, Maribel Miguel-Pérez, Anders Fasth

**Affiliations:** 1 Department of Pediatrics, Skåne University Hospital, Lund University, Sweden; 2 Department of Diagnostic Imaging, Gildhøj Private Hospital, University of Copenhagen, Denmark; 3 Department of Pediatrics, Rigshospital, University of Copenhagen, Denmark; 4 Department of Diagnostic Imaging, Rigshospital, University of Copenhagen, Denmark; 5 Human Anatomy and Embryology Unit, Department of Experimental Pathology and Therapeutics, Faculty of Medicine (CHS Bellvitge) University of Barcelona, Spain; 6 Department of Pediatrics, University of Gothenburg, Sweden

## Abstract

**Background:**

The presence of enthesitis (insertional inflammation) in patients with juvenile idiopathic arthritis (JIA) is difficult to establish clinically and may influence classification and treatment of the disease. We used ultrasonography (US) and color Doppler (CD) imaging to detect enthesitis at the small and deep-seated proximal insertion of the *gluteus medius *fascia on the posterior iliac crest where clinical diagnosis is difficult. The findings in JIA patients were compared with those obtained in healthy controls and with the patients' MRI results.

**Methods:**

Seventy-six proximal *gluteus medius *insertions were studied clinically (tenderness to palpation of the posterior iliac crest) and by US and CD (echogenicity, thickness, hyperemia) in 38 patients with JIA and in 38 healthy controls, respectively (median age 13 years, range 7-18 years). In addition, an additional MRI examination of the sacroiliac joints and iliac crests was performed in all patients.

**Results:**

In patients with focal, palpable tenderness, US detected decreased echogenicity of the entheses in 53% of the iliac crests (bilateral in 37% and unilateral in 32%). US also revealed significantly thicker entheses in JIA patients compared to healthy controls (p < 0.003 left side, p < 0.001 right side). There was no significant difference in thickness between the left and right sides in individual subjects. Hyperemia was detected by CD in 37% (28/76) of the iliac crests and by contrast-enhanced MRI in 12% (6/50).

**Conclusions:**

According to US, the *gluteus medius *insertion was thicker in JIA patients than in controls, and it was hypoechoic (enthesitis) in about half of the patients. These findings may represent chronic, inactive disease in some of the patients, because there was only limited Doppler flow and MRI contrast enhancement. The present study indicates that US can be useful as an adjunct to clinical examination for improved assessment of enthesitis in JIA. This may influence disease classification, ambition to treat, and choice of treatment regimen.

## Background

Enthesopathies include affections of the sites where tendons, ligaments, capsules, or fascia are attached to bone (i.e., the entheses), and they are either of mechanical origin (overuse or trauma) or occur secondary to inflammatory disease. The pathogenesis of enthesitis is not fully understood, although an interaction of microtrauma at disease sites and infectious agents in genetically susceptible individuals has been proposed [1]. The most commonly affected sites are the calcaneal insertions of the Achilles tendon and the plantar fascia [2,3]. Furthermore, there is frequently an impact on the patella, the greater trochanter, the iliac crest, and the ischial tuberosity, and in children also on different regions of the foot (e.g., the tarsal area) [1,4].

Juvenile idiopathic arthritis (JIA) is a heterogeneous set of conditions linked by the common feature of arthritis lasting for at least 6 weeks in a child younger than 16 years [5]. According to the ILAR classification JIA patients are divided into 7 subgroups and enthesitis is seen primarily in the enthesitis related arthritis subgroup (ERA) [5], a form of undifferentiated spondyloarthropathy (SpA) [6]. Enthesitis can be located in the axial or peripheral skeleton, and it is the main cause of problems in JIA classification, because the afflicted patients are assigned to more than one subgroup [7].

A diagnosis of enthesitis is difficult to make, because it is based on palpable tenderness at insertion sites alone [8]. Superficial entheses such as the insertion of the Achilles tendon can exhibit soft tissue swelling, which is in contrast to insertions of the plantar fascia and deep-seated entheses at certain other locations (e.g., the iliac crest) [9-11]. Enthesopathies can occur at numerous anatomical sites but are more common in the weight-bearing lower limbs in both adults and children [1,12-14].

Plain radiography is mainly capable of visualizing the bony part of an enthesis, and thus reveals only the late stages of disease, which can include enthesophytes, bone erosions, or soft tissue calcifications [15]. The early signs of inflammatory enthesitis involve the soft tissues and can be demonstrated by MRI or ultrasonography (US), as has been shown in adults with SpA [16-18].

In the present study, we assessed US and color Doppler (CD) for detection of enthesitis at the small and deep-seated proximal insertion of the *gluteus medius *fascia on the posterior iliac crest in JIA patients, and compared the findings with those obtained in healthy controls and with the patients' MRI results.

## Methods

This study was conducted over a period of 2.5 years at the Department of Pediatrics (Rigshospital) of the University of Copenhagen, Denmark. It was approved by the local research ethics committee (Videnskabsetiske Komiteer for Region Hovedstaden), and informed consent was obtained from all parents and children over 13 years of age.

### Patients and healthy controls

The study included 38 consecutive patients at the Department of Pediatrics in Copenhagen. The patients were 7-18 years of age and presented with subjective pain in the buttocks and had tenderness on palpation of the posterior iliac crest (n = 76). US examination including CD and a contrast-enhanced MRI were performed on all patients. Thirty-eight healthy age- and sex-matched controls were assessed clinically for tenderness on palpation of the posterior iliac crest and by Doppler-US; these evaluations were done at the Department of Pediatrics, Skåne University Hospital, Lund, Sweden, using the same protocols and the same type of US equipment as for the patients in Denmark. After the patient cohort had been established, the controls were recruited among families of hospital staff members and among patients with no history of arthritis or chronic pain.

### Clinical assessment

Patients who had previously been diagnosed with JIA on the basis of the revised criteria of the International League of Associations for Rheumatology (ILAR, 2004) [5] were examined by one of two experienced pediatric rheumatologists for tenderness on palpation of the posterior iliac crest. Tenderness was assessed by applying digital, focal pressure on the proximal insertions of *gluteus medius *at the iliac crest. The following clinical variables were recorded: duration of disease, duration of pain according to the patient/parents, HLA-B27, and pharmacological treatment. HLA-B27 positivity was analyzed at different laboratories using commonly employed methods. Since results of the different methods for analysis of HLA-B27 are insignificant, we have not stressed this further [19].

### US and CD assessment

**Figure 1:**
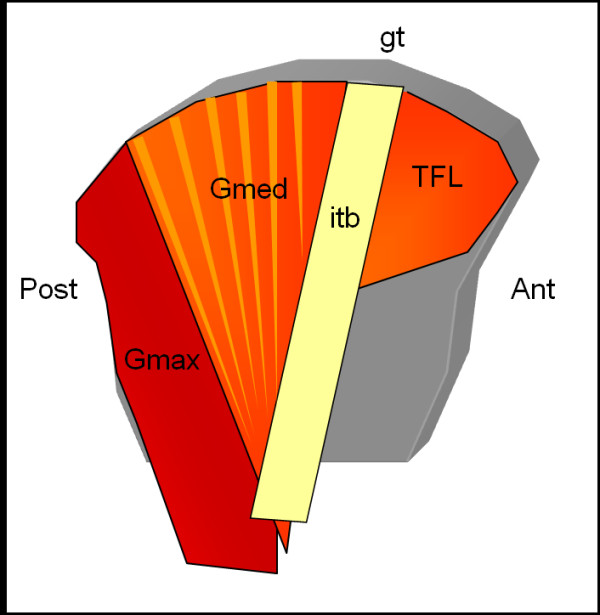
**Schematic, lateral view of the iliac bone**. The fascia of the *gluteus medius *muscle (Gmed) inserts proximally on the posterior aspect of the iliac crest, dorsally to the insertion of the iliotibial band (itb) on the gluteal tubercle (gt) and anterior to the insertion of the *gluteus maximus *muscle (Gmax). TFL = *tensor fascia latae *muscle.

On the same day as the clinical assessment an US examination was performed, by a radiologist specialized in musculoskeletal US since 20 years and with clinical and scientific experience in color Doppler examination in rheumatology. The machine was a Logiq 9 scanner, GE Healthcare (Chalfont St. Gilles, UK) equipped with a 13-5 MHz linear transducer. The posterior iliac crests were examined with the subject lying supine on the right and the left side, respectively. The proximal *gluteus medius *inserts posteriorly on the iliac crest, posterior to the insertion of the iliotibial band on the gluteal tubercle and anterior to the insertion of the *gluteus maximus *(Figure 1). The *gluteus medius *is covered by fascia, and its muscular fibers also insert on the outer surface of the ilium between the posterior and anterior gluteal lines.

**Figure 2:**
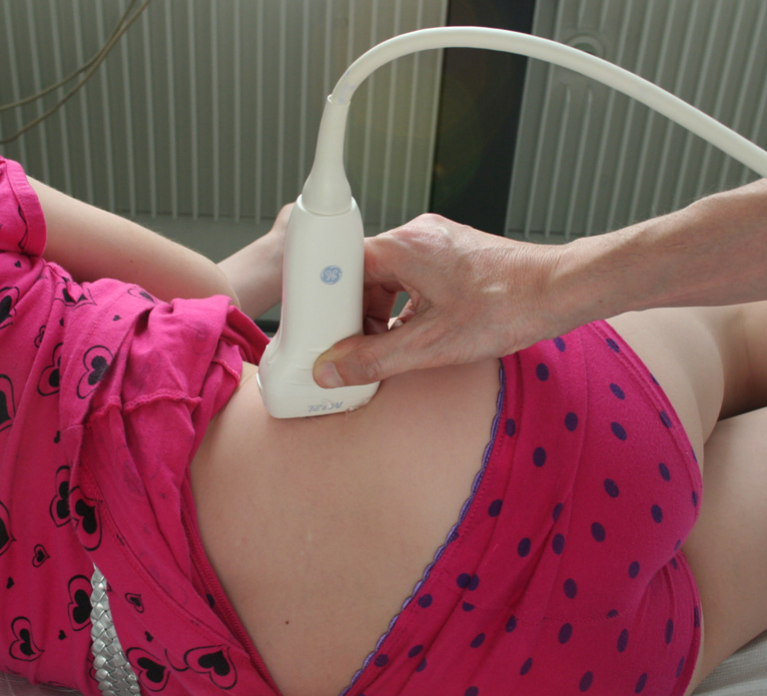
**Longitudinal US scanning of the proximal insertion of the *gluteus medius *fascia on the posterior iliac crest in a child lying on the side**.

**Figure 3:**
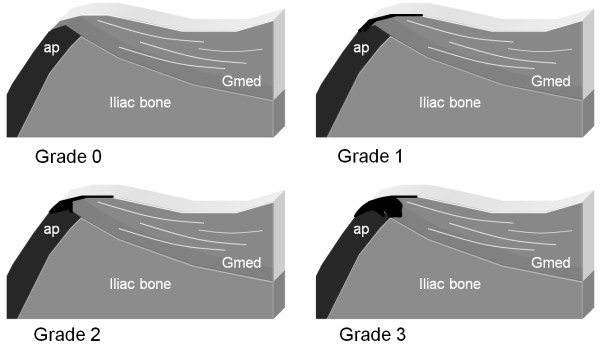
**Schematic view of different grades of enthesopathy of the *gluteus medius *insertion as presented in longitudinal US scans**. Gmed = *gluteus medius *muscle, ap = apophysis. Grade 0: normal enthesis. The fascia is a thin echorich line. Grade 1 enthesopathy: hypoechoic fascial thickening at the insertion. Grade 2 enthesopathy: small cranial triangular hypoechoic area in the muscle between fascia and iliac crest. Grade 3 enthesopathy: large hypoechoic area in the muscle with caudal extension.

**Figure 4:**
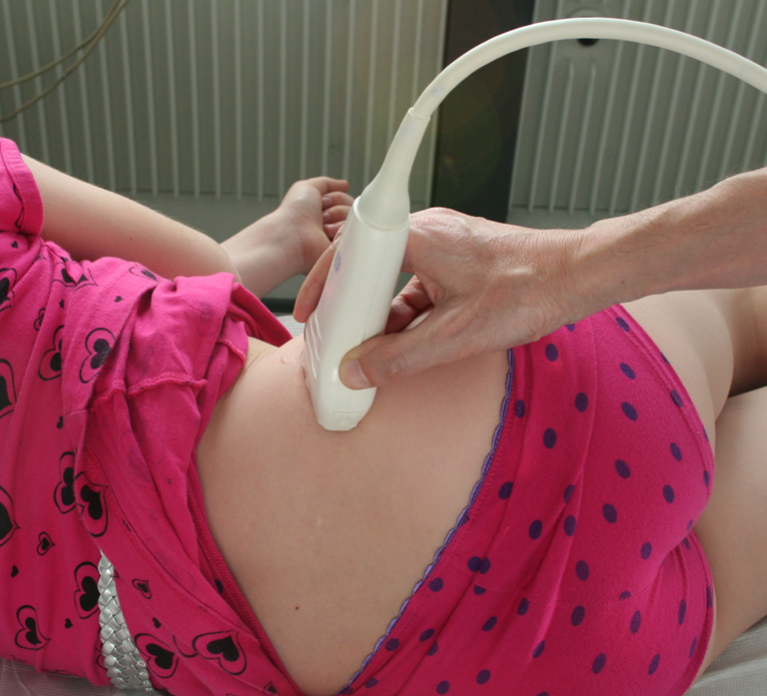
**Transversal US scanning of the proximal insertion of the *gluteus medius *fascia on the posterior iliac crest in a child lying on the side**.

In each patient, an oblique longitudinal US scanning of the gluteal insertion (Figure 2) was obtained perpendicular to the iliac crest, and this was done to search for hypoechoic thickening of the fascia, hypoechoic areas in the muscle insertion, or irregularities of the bony surface. Hypoechoic changes were graded from 0 to 3 (Figure 3): grade 0, normal enthesis; grade 1, hypoechoic fascial thickening at the insertion; grade 2, small cranial triangular hypoechoic area in the muscle between the fascia and iliac crest; grade 3, large hypoechoic area in the muscle with a caudal extension. Grades 1-3 were considered pathological signs of enthesopathy. The thickness of the insertion was measured perpendicularly to the iliac crest at the level of the physis, and this was done using a technique similar to that applied to measure the thickness of the *supraspinatus *tendon at the level of the humeral neck [20]. The findings of the CD examination were assessed as presence or absence of hyperemia. The US examination was completed by an oblique transverse scan of the gluteal enthesis (Figure 4).

### Contrast MRI assessment

The patients' sacroiliac joints and iliac crests were examined by MRI, and the results were described by a radiologist specialized in musculoskeletal MRI. Due to limited availability, it was necessary to use two different MRI systems (both version B17): a Siemens Avanto TIM 1.5 Tesla unit in 20 patients, and a Siemens Magnetom Trio TIM 3.0 Tesla unit in 18 patients. The following MRI sequences were performed: coronal oblique and axial oblique T1w sequences; coronal oblique and axial oblique STIR sequence (1 patient) and/or fat-suppressed T2w sequences (37 patients); coronal oblique and axial oblique fat-suppressed T1w sequences after injection of intravenous contrast (Dotarem 0.1 mmol/kg, 25 patients). The sacroiliac joints were examined for sacroiliitis (thinning, irregularity, or ankylosing of the joint lines), bone destruction, subchondral edema (hyperintense areas on STIR and fat-suppressed T2w sequences, and hypointense areas on T1w sequences), subchondral fatty deposits (hypointense areas on STIR and fat-suppressed T2w sequences, and hyperintense areas on T1w sequences), subchondral fibrosis (hypointense areas on STIR, fat-suppressed T2w sequences, and T1w sequences), and synovial or subchondral contrast enhancement (fat-suppressed T1w sequences). The iliac crests were examined for bone edema (pathological hyperintense areas on STIR and fat-suppressed T2w sequences, and hypointense areas on T1w sequences), fatty deposits (hypointense areas on STIR and fat-suppressed T2w sequences, hyperintense areas on T1w sequences) or subchondral fibrosis (hypointense areas on STIR, fat-suppressed T2w sequences, and T1w sequences). The iliac crest and the insertion of the gluteal fascia and muscle were examined for contrast enhancement (fat-suppressed T1w sequences).

### Cadaveric specimens

Anatomical confirmation of the US findings in healthy controls was obtained by analyzing dissection specimens from 5 adult human cadavers.

### Statistical analysis

Statistical analyses were performed using SPSS version 16 (SPSS Inc., Chicago, IL, USA). For all calculations, p < 0.05 was considered statistically significant. Differences between patients and controls were analyzed using the paired t-test.

## Results

### Clinical findings in patients and healthy controls

Our study included a total of 38 JIA patients with subjective pain in the buttocks and palpable tenderness at the proximal insertion of the gluteal muscles on the iliac crest. Demographic features, clinical and laboratory assessments, and treatment of the patients are listed in Table 1. Twenty-seven had ERA (71%; 12 boys, 15 girls), 5 poly-JIA (13%; 1 boy, 4 girls), 4 psoriasis arthritis (10%; 2 boys, 2 girls), 1 systemic JIA (3%; 1 girl), and 1 oligo-JIA (3%; 1 girl). Fourteen (37%) of the patients (47% of the boys, 30% of the girls) were HLA-B27 positive. Thirty-five (92%) of the 38 JIA patients reported having bilateral pain in the buttocks. The remaining 3 (8%) had unilateral pain, and those individuals had previously experienced pain on the contralateral side. At inclusion, all 38 patients had bilateral tenderness on palpation (76 iliac crests), by comparison, the 38 age- and sex-matched controls had no pain or palpable tenderness at the proximal insertion of the gluteal muscles on the iliac crest.

**Table 1:** Clinical findings in 38 JIA patients with 76 symptomatic posterior iliac crests

Characteristic	Number (%)	Median	Range
			
Sex			
Male	15 (39%)		
Female	23 (61%)		
Subgroups			
Enthesitis related arthritis	27 (71%)		
RF-negative polyarthritis	5 (13%)		
Psoriasis arthritis	4 (10%)		
Systemic	1 (3%)		
Oligoarthritis	1 (3%)		
Age, years		13	7-18
Disease duration, months		26	0-116
Duration of focal pain, months		6.5	1-48
Iliac crests (n = 76) with palpable tenderness	76 (100%)		
HLA B27 positive, number of patients	14 (37%)		
Second-line drug therapy			
Methotrexate	7 (22%)		
Sulphasalazine	4 (13%)		
Biologic therapies and Methotrexate			
Adalimumab	8 (26%)		
Etanercept	5 (16%)		
Infliximab	4 (13%)		
Systemic corticosteroids and Methotrexate	3 (10%)		

### US findings in healthy controls and patients

**Figure 5:**
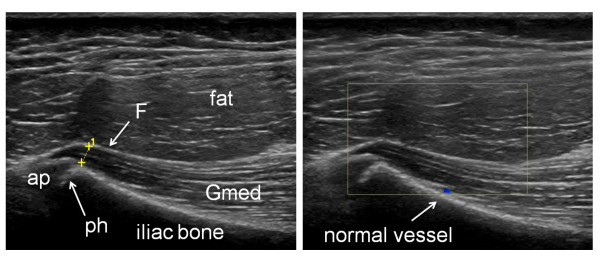
**Longitudinal US scanning of the proximal enthesis of the *gluteus medius *fascia on the posterior iliac crest in a 13 year old healthy control**. A) The insertion is measured (1) perpendicularly to the iliac crest at the level of the physis (ph). The *gluteus medius *(Gmed) fascia (F) is hyperechoic and thin. The apophysis (ap) is cartilaginous. B) No hyperemia is seen on longitudinal CD examination.

**Figure 6:**
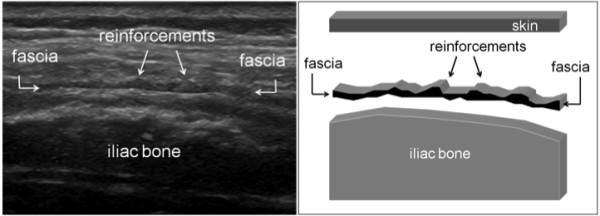
**Transversal US scanning of the *gluteus medius *fascia in a healthy control**. A) The wavy aspect of the gluteal fascia caused by the longitudinal reinforcements. The fascia is hypoechoic due to the anisotropic artifact. B) Schematic view of longitudinal fascia reinforcements in a transversal US scan.

**Figure 7:**
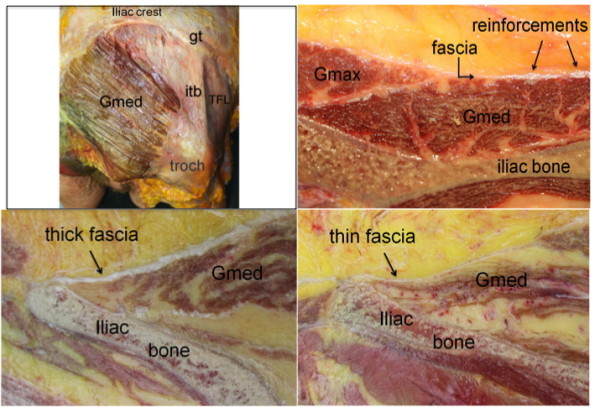
**The normal *gluteus medius *muscle and fascia in an adult cadaveric specimen**. A) Lateral view showing the longitudinal fascia reinforcements over the *gluteus medius *muscle (Gmed). The iliotibial band (itb) inserts on the gluteal tubercle (gt) of the iliac crest and covers the greater trochanter (troch). TFL = *tensor fascia latae *muscle. The *gluteus maximus *muscle is resected. B) Transversal cut of the Gmed insertion. Gmax = *gluteus maximus*. C) Longitudinal cut of the Gmed insertion at the level of a fascia reinforcement. D) Longitudinal cut of the Gmed insertion between fascia reinforcements.

**Figure 8:**
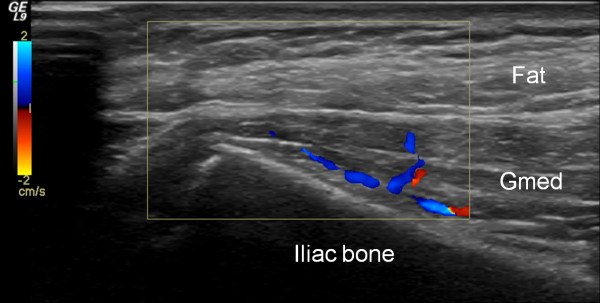
**Longitudinal CD examination showing normal arteries (blue) and veins (red) between the iliac bone and the *gluteus medius *muscle (Gmed) with collateral perforants extending through the muscle**.

US examination of the 38 healthy controls revealed normal anatomy of the posterior iliac crests bilaterally (n = 76) (Figure 5A). The apophyses were cartilaginous and without any visible ossification center. The thickness of the apophysis (measured from the edge of the crest to the epiphysis) decreased with increasing age of the subjects, from 7.5 mm at 7 years to 4 mm at 18 years. The US aspect of the proximal *gluteus medius *insertions of the controls was normal in 75 iliac crests and pathological in one (hypoechoic changes, grade 2). In all controls, US showed regularly distributed longitudinal reinforcements of the fascia with a wavy appearance in transverse scanning planes (Figure 6A-B). These reinforcements could also be visualized in the adult cadaveric dissection samples analyzed (Figure 7A-D). The thickness of the *gluteus medius *insertion, measured by US, was 1.0-3.3 mm (median and mean 1.9 mm, Table 2). There was no significant difference between the left and right sides in individual subjects (Table 2). CD detected no vascularization in any of the *gluteus medius *insertions or in the iliac crest cartilage in the controls (Figure 5B). Normal arterial and venous supply was seen as regularly distributed parallel vascular branches that originated from the deep branch of the superior gluteal artery [21] perpendicular to the crest of the iliac bone and the *gluteus medius *muscle (Figure 8).

**Table 2:** US measurements of the thickness of the *gluteus medius *insertion on 76 iliac crests in 38 patients with JIA and on 76 iliac crests in 38 healthy controls.

	Left		Right		
	**Mean, 2SD (mm)**		**Mean, 2SD (mm)**		

Patients	2.47	1.92	2.63	2.21	p* < 0.193

Controls	1.90	0.91	1.99	0.86	p* < 0.206

	p* < 0.003		p* < 0.001		

* Paired t-test

**Figure 9:**
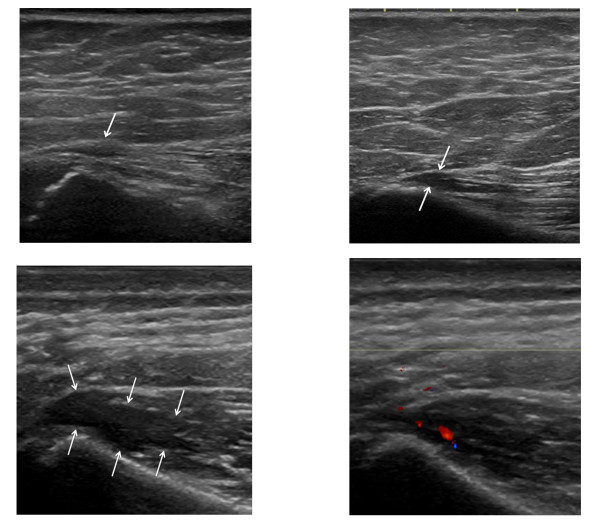
**Longitudinal US scanning of *gluteus medius *enthesopathy in patients with JIA**. A) Grade 1: hypoechoic fascial thickening at the insertion (arrow). B) Grade 2: small cranial triangular hypoechoic area in the muscle between fascia and iliac crest (arrows), here with a thin caudal extension. C) Grade 3: large hypoechoic area in the muscle with caudal extension (arrows). D) Longitudinal CD examination shows hyperemia at the insertion.

US examinations of the 76 proximal *gluteus medius *insertions of the patients with JIA revealed various pathological hypoechoic changes (grades defined in the methods section) in longitudinal scanning planes of 40/76 crests (53%, Table 3). The changes were of grades 1, 2, and 3 in 5 (13%), 8 (20%), and 27 (67%) of the crests, respectively (Figure 9A-C), and they were bilateral in 14 patients, unilateral in 12, and absent in 12. The *gluteus medius *insertion was considered normal (grade 0) in 36 crests (47%). The *gluteus medius *insertion was 0.5-5.2 mm thick (median and mean 2.5 mm), which was thicker than observed in the healthy controls. There was no significant difference between the left and right sides in individual patients (Table 2) and the thickness of the gluteus medius insertion and the patients' age displayed no correlation. Data was tested for normality and found to be normally distributed. There was no difference in Doppler-US findings in HLA-B27 positive and HLA-B27 negative patients.

**Table 3:** Morphological changes on US and hyperemia on color Doppler of the *gluteus medius *insertion on the iliac crest in 38 patients with JIA and in 38 healthy controls.

	Iliac crest patients (n = 76)	Iliac crest controls (n = 76)
Hypoechoic changes		
grade 0	36	75
grade 1	5	1
grade 2	8	0
grade 3	27	0

CD flow	28	0

**Figure 10:**
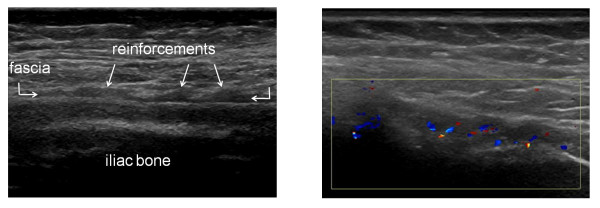
**Transversal US scanning of the *gluteus medius *fascia insertion in a patient with JIA and enthesopathy**. A) The wavy aspect, due to the longitudinal reinforcements of the fascia, is visualized in the thickened fascia. B) Transversal CD examination shows hyperemia at the insertion.

On transverse scanning planes, the longitudinal reinforcements of the gluteal fascia that created a wavy appearance were preserved and showed increased thickness (Figure 10A), and hyperemia was observed in some patients (Figure 10B). CD detected signs of hyperemia in 28/76 iliac crests (37%; Table 3 Figure 9D). The structure of the cartilaginous apophysis of the posterior iliac crest was similar in patients and controls. Furthermore, there were no apparent irregularities of the bony surfaces in any of the subjects.

### MRI findings in patients

Ten patients had already had an MRI examination of the sacroiliac joints and iliac crests at a median of 7.5 weeks (range 2-29 weeks) before they were included in the study and examined by US. Due to limited availability of MRI scanners, only 4 patients underwent US and MRI examination in the same week. In 24 of the patients, MRI was performed after US (median 4 weeks, range 2-19 weeks).

**Figure 11:**
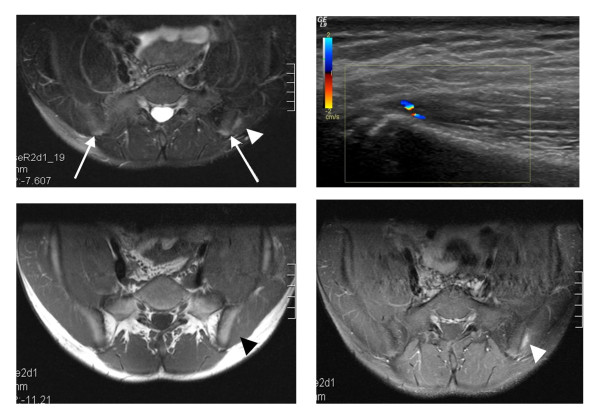
**US and MRI images of a 15 year old boy with JIA and enthesitis**. A) Fat suppressed T2-weighted MRI sequence showing bilateral, physiological edema of the iliac crests (arrows). Focal muscle edema at the left *gluteus medius *insertion (arrowhead) indicates enthesopathy. B) Longitudinal US with CD of the left insertion showing a hypoechoic area and hyperemia. C) T1-weighted MRI sequence before intravenous gadolinium contrast injection. D) Fat suppressed T1 sequence after contrast injection showing enhancement (arrowhead) in the muscular insertion.

MRI detected signs of enthesitis in 12 iliac crests. Iliac crest bone edema was considered pathological in 6 of the 76 crests (8%, 3 patients). Contrast media was offered to all patients, 25 accepted and 13 declined. Contrast enhancement was demonstrated in the fascial and/or muscular insertion in 6/50 iliac crests (12%, 5 patients) but not in the cartilage or the bone marrow of the crests (Figure 11A-D). In only one of the 6 crests with contrast enhancement on MRI, hyperemia was also detected on CD; the time span between Doppler-US and MRI in this case was 2 weeks. Bone edema and contrast enhancement never occurred in the same iliac crest.

MRI showed that 16 of the patients had sacroiliitis (28 sacroiliac joints); 14 (88%) of those individuals had ERA, 1 had poly-JIA, and 1 had oligo-JIA. In the patients with sacroiliitis, the median duration of disease was 20 months (range 0-116 months), the median duration of focal pain was 12 months (range 1-48 months), and 6 of these subjects (38%) were HLA-B27 positive. In 10 of the patients with sacroiliitis on MRI (14 sacroiliac joints), US detected enthesitis at the iliac crest.

## Discussion

SpA is characterized by a higher frequency of extra-axial symptoms in children than in adults [2], and peripheral enthesitis in JIA often precedes the symptoms of axial involvement, in association or not with peripheral arthritis [22]. In JIA, the pain caused by peripheral inflammatory enthesitis can be severe, disabling, and persistent. Nevertheless, due to diagnostic uncertainty, enthesitis is not always recognized and hence may not be treated appropriately [14,23-27].

The diagnosis of enthesopathy is traditionally based on palpable tenderness at insertion sites [8]. Site-specific tenderness of various origins (e.g., post-traumatic, psychosomatic, inflammatory, and transient growing pains) is common in children [28,29]. The prevalence of enthesitis in the healthy population is unknown [3]. Deep-seated entheses (e.g., on the iliac crest) are difficult to palpate and when entheseal insertions are close to the joint line (e.g., the patellar ligament and the quadriceps tendon) it may be difficult to attribute palpable pain to enthesitis or joint synovitis [30]. Furthermore, apophysitis (e.g., Osgood-Schlatter disease) [31,32], osteonecrosis (e.g., Legg-Calvé-Perthes disease), epiphysiolysis, coxitis simplex, or infection may in some cases simulate enthesis involvement in children and adolescents.

In patients with ERA, it has been shown that the number of active entheses and joints at onset can predict sacroiliitis at follow-up [33]. Inflammatory back pain is rarely present at onset of JIA, but sacroiliac and spinal involvement occurs in up to two-thirds of children within 10 years of disease onset [25,34,35]. Sacroiliitis often remains clinically unrecognized [36], and, once it has developed, anti-inflammatory therapies cannot always prevent progression of the condition [37-39]. These observations suggest that it is important to implement early diagnosis and therapy of enthesitis in order to alter the course of the disease.

The present study is descriptive and was not designed to compare results from clinical and US assessments. The inclusion criterion was JIA and palpable tenderness at the posterior iliac crest, a part of the routine investigation of JIA patients at the Pediatric Rheumatology Outpatient Clinic, Rigshospitalet, Copenhagen. Patients without focal clinical symptoms were not presented to the US examiner, who was blinded to other aspects of the clinical status, subtype etc of the children. The same examiner performed the US examinations on patients and healthy controls and was not blinded to whether he was investigating JIA patients or healthy controls, which could be a bias.

Using the ILAR criteria for defining disease, our study shows that enthesitis can occur in other JIA subgroups than ERA as well (Table 1). We also noted that slightly more girls than boys were diagnosed with ERA, which does not agree with reports indicating that ERA usually affects boys more often than girls [23]. The reason for the predominance of girls in our study, as well as in a recent retrospective investigation comparing the ILAR and Amor criteria for SpA in children [36], is not clear.

Nearly two decades ago, Lehtinen et al. [30] were the first to demonstrate that US could detect abnormalities associated with lower limb enthesitis in patients with SpA. Later studies have also shown that US is more sensitive than clinical assessment for diagnosing enthesitis in adult SpA patients [40-42] and that Doppler-US is a sensitive method for detecting abnormal blood flow in and around peripheral entheses in adults with SpA [43-47]. In 2005, the Outcome Measures in Rheumatology (OMERACT) network of working groups introduced the following definition of the US signs of enthesopathy: an abnormally hypoechoic and/or thickened tendon or ligament at its bony attachment seen in 2 perpendicular planes that may exhibit Doppler signal and/or bony changes [48]. Other recognized US signs include focal or diffuse loss of normal tendon or ligament fibrillar structure, intratendinous or intraligamentary calcifications, bone erosions, new bone formation (enthesophytes), and associated abnormalities of adjacent bursae [42,49].

The first MRI studies of peripheral joint involvement in SpA emphasized the extrasynovial nature of the inflammatory process, commonly enthesitis, and showed that this included peri-entheseal soft tissues and bone marrow adjacent to entheseal insertions [12,50]. The MRI patterns of SpA enthesitis have the following characteristics: edema in the adjacent bone and the surrounding soft tissues, with a high signal on STIR or fat-suppressed T2w sequences, and a low signal on T1w sequences [18,50,51]; enhancement of the signal in the soft tissue part of the enthesis on fat-supressed T1w sequences after intravenous injection of gadolinium contrast [52].

US has been reported to be more sensitive than MRI for early detection of soft tissue involvement in Achilles tendon enthesitis [18]. Also compared to MRI, US has the general advantages of being cheaper, mobile, instantly accessible at bedside, non-invasive, and easy to combine with the clinical assessment (interactivity), and it does not require sedation of young children [53,54]. In addition, US allows assessment of multiple locations during the same session. Modern high-frequency US transducers provide images with unsurpassed resolution for examination of superficial musculoskeletal structures in children. On the other hand, US does not give a complete picture of all joint structures, because the ultrasound beams cannot penetrate bone. Some areas are hidden by overlying bony structures, such as axial ligament insertions in the spine. For the same reason bone edema is not detectable by US. The main disadvantages of MRI are the high cost of the equipment and that it is frequently inaccessible.

In adults with SpA, US and MRI have been shown to be more sensitive than clinical examination for detecting peripheral enthesitis in the limbs (early signs of inflammation and signs of structural damage) [40,42,44,47,51,55,56]. It is not yet clear what role these imaging methods can play in assessment of enthesitis in JIA, because thus far the pediatric literature contains only one case report [57] and one investigation comparing US and clinical examination [58]. Our study is the first to concern US and MRI examinations performed on JIA patients to detect enthesitis in a small and deep-seated insertion, a situation in which clinical diagnosis is difficult and imaging would be of great importance.

Enthesopathy at the iliac crest can be of mechanical origin (chronic overuse or acute trauma) or arise secondary to inflammatory disease. Mechanical enthesopathy has been found to occur in both adolescents [59] and adults [60,61] who participate in sports, primarily in running, and the changes that were observed in the cited investigations were localized to the anterior part of the iliac crest, and were visualized by MRI, US, and scintigraphy [59-62]. In our study, US signs of inflammatory enthesitis were demonstrated at the insertion of the *gluteus medius *muscle on the posterior aspect of the iliac crest. It is plausible that the posterior location of enthesitis in our JIA patients can be explained by the important role that the *gluteus medius *plays in maintaining normal postural control and hip joint motion during walking [63-65]. Peripheral inflammatory enthesitis is more frequently found in the weight-bearing lower limbs in both adults and children [1,12-14]. The reason for this is not clear, although recent studies have suggested an association between mechanical factors and inflammatory disease [66,67].

Normal and pathological US features have been described for the distal insertion of the *gluteus medius *tendon on the greater trochanter (tendinopathy and tears) [68] but never for the proximal insertion of this muscle. It is important that US appearance of the longitudinal reinforcements of the normal *gluteus medius *fascia are well recognized in order to avoid any confusion with signs of enthesitis. Our patients, who had hypoechoic entheses on US, had the same regularly distributed fascial reinforcements (Figure 10A), which suggests that there is a predisposition to fascial inflammation in these areas of reinforcement. To avoid diagnostic errors in differentiating normal and inflamed entheses, it is also essential to use a meticulous scanning technique that allows clear interpretation of possible anisotropic artifacts that can easily occur at the level of the entheseal insertion where fibers are curved towards the iliac crest.

Doppler activity was detected in only 37% of the iliac crests in patients with JIA. This suggests either poor sensitivity of the CD technique, possibly due to the deep location of the iliac crest insertion, and/or that a great number of the detected US anomalies were associated with chronic inactive disease. In our study we also displayed the normal US aspect of the posterior iliac crest of children, with a peripheral cartilaginous apophysis on the bony margin of the ilium at the level of the epiphysis (Figure 5A). No secondary ossification center was observed in any of the patients or controls, which can be explained by the fact that ossification usually appears in the anterior part of the iliac crest at the age of approximately 13-15 years [59]. As a child matures, gradual ossification of cartilage occurs in a posterior direction until the age of 25 years [59].

In a small MRI pilot study of the iliac crest in healthy young individuals we found that the physiological edema associated with normal epiphyseal growth was impossible to distinguish from pathological edema caused by enthesitis. This has been demonstrated in previous studies, at the iliac crest [69] and at the wrist [70], and is the reason why MRI of the control subjects was not performed in our study. Consequently, it may be difficult to use MRI to detect any pathological edema caused by enthesitis in children and adolescents. The 38 MRI examinations we conducted revealed signs of enthesitis in only 12 iliac crests in 8 patients. These MRI examinations were performed as routine scannings to detect sacroiliitis (matrix, orientation of scanning planes) and not optimized for imaging of small entheseal insertions on the iliac crest. Because of low spatial resolution the sensitivity of MRI is decreasing with the size of the enthesis being examined. In our study, the detailed anatomical structures of the small soft tissue part of the *gluteus medius *enthesis that were detected by US were not visualized by MRI; this might have occurred because MRI mainly reveals edema in cartilaginous and bony parts, and hence this method is less sensitive to the changes seen in the soft tissue portion of the enthesis in enthesitis [71,72]. Furthermore, the discrepancies we observed between the US and MRI findings might have been related to the time span between the two examinations in our study and to the fact that a number of patients received pharmacological treatments with a potential effect on enthesitis. MRI examinations were performed on the same day as US in only four patients, and thus the MRI results for those subjects represented the only true control examinations; those MRI examinations showed no signs of enthesitis or any contrast enhancement.

A weakness of the present study is that only one experienced musculoskeletal radiologist did evaluate the accuracy of the enthesitis US examination.

## Conclusion

In our study, US showed that the 76 symptomatic *gluteus medius *insertions in the 38 patients with JIA were thicker than the corresponding asymptomatic insertions in the healthy controls, and they were hypoechoic (enthesitis) in about half of the patients. The US findings in some of the cases may have indicated chronic, inactive disease, since there was limited Doppler flow and MRI contrast enhancement. The present observations suggest that using US as an adjunct to clinical examination can improve assessment of enthesitis in JIA, and this may influence disease classification, ambition to treat, and the choice of treatment regimen.

## Competing interests

The authors declare that they have no competing interests.

## Authors' contributions

LL participated in design of the study, performance of ultrasound and clinical examinations, acquisition of data, statistical analysis and was responsible for analysis of the results, and drafting of the manuscript. MCP was involved in design of the study, analysis of the results, performance of ultrasound examinations, and drafting of the manuscript. SN helped design the study, perform clinical examinations, and revise the manuscript. MZ contributed to design the study, performance of clinical examinations, and revision of the manuscript. CT helped design the study, analyze the results, and perform MRI examinations. MMP took part in design of the study, performance of anatomical dissections, and revision of the manuscript. AF helped design the study, analyze the results, and draft the manuscript. All authors read and approved the final manuscript.
